# COVID-19: Mythe ou réalité ? Note sur le cas de la ville de Bossangoa (République centrafricaine)

**DOI:** 10.48327/mtsi.2021.165

**Published:** 2021-10-29

**Authors:** Sosthène Constant SABE

**Affiliations:** Hôpital régional universitaire de Bossangoa, République centrafricaine

**Keywords:** Covid-19, Bossangoa, République centrafricaine, Afrique subsaharienne, Covid-19, Bossangoa, Central African Republic, Sub-Saharan Africa

## Abstract

En mai 2021, les infections par la Covid-19 sont présentes à Bossangoa, RCA, avec une faible incidence et une faible morbidité. La population les assimile à une pathologie locale considérée comme banale et ne respecte pas les mesures barrières décidées par les autorités du pays.

## Abstract (en)

In May 2021, Covid-19 infections are present in Bossangoa, Central African Republic, with an apparent low incidence and a low morbidity. The population equates them with a local pathology considered trivial and does not respect the barrier measures decided by the country's authorities.

L’épidémie d'infection par le virus SARS-CoV2 est apparue en Chine en décembre 2019. À partir de février 2020, de nombreux cas sont observés dans le nord de l'Italie puis dans tous les pays d'Europe. L’épidémie y a pris une tournure plus agressive qu'attendue avec une mortalité élevée et de grandes répercussions sur la vie et le travail de chacun.

Un premier cas de Covid-19 a été identifié en République centrafricaine (RCA) le 8 mars 2020. Cependant un voyageur a possiblement été infecté plus tôt, dès la fin février, soit dans ce pays, soit au cours d'un vol de retour passant par le Cameroun [1]. Dans le même temps, à Bossangoa, ville située à 270 km au nord de Bangui, capitale de la RCA, la population n'est pas ou peu consciente de cette pandémie et ne respecte pas les mesures barrières définies par les autorités.

Depuis que le ministère de la Santé et de la Population a déclaré l’épidémie en RCA le 14 mars 2020, la ville de Bossangoa, chef-lieu de la préfecture de Ouham, peuplée d'environ 180 000 habitants, reste épargnée malgré quelques cas positifs de Covid-19 dépistés au début de l’épidémie au point de contrôle situé au kilomètre 26 de Bangui. Certains de ces cas étaient en provenance de Bossangoa. Les autorités politico-administratives, sanitaires et les organisations humanitaires de la localité ont alors déclenché des séries de campagnes de sensibilisation à travers les médias, des séances de communication éducative avec différents groupes sociaux, avec les relais communautaires, des distributions de prospectus sur les symptômes de la maladie et sur les mesures barrières. Un comité préfectoral de gestion des épidémies et catastrophes a été mis en place. Cependant, la population de Bossangoa reste sceptique, car pour elle, les symptômes de la Covid-19 ne sont autres que ceux d'une maladie qui a déjà sévi dans le passé dans cette ville. Cette maladie, dénommée *dong kilo* en gbaya, la langue locale, est un symptôme grippal saisonnier avec toux, fièvre, écoulement nasal. Elle est souvent traitée avec une décoction de feuilles et tiges d'une plante herbacée qui pousse au bord des cours d'eau, associée à des antipyrétiques comme le paracétamol.

Un premier cas de Covid-19 a été identifié le 10 mars 2021 à l'hôpital régional universitaire de Bossangoa sur un échantillon de crachat recueilli pour la recherche de bacille de Koch. Un prélèvement nasopharyngé a confirmé le cas par un test RT-PCR. Jusqu'au 1^er^ mai 2021, 38 cas positifs ont été identifiés parmi lesquels nous avons des sujets symptomatiques et les sujets contacts qui sont asymptomatiques. Après 21 jours de traitement, 18 patients sont guéris avec un contrôle PCR négatif, 19 sont encore sous traitement et un patient est transféré à Bangui après trois contrôles dont le résultat reste toujours positif.

Malgré la présence avérée de la maladie dans la ville, on ne ressent pas en mai 2021 de crainte dans la communauté. Les mesures barrières ne sont pas respectées. Les contacts des cas deviennent à leur tour des cas positifs sans inquiéter la population de Bossangoa. Il n'y a pas la perception d'un risque infectieux nouveau.

En conclusion, la Covid-19 présente apparemment un faible taux d'incidence dans la région de Bossangoa et elle n'entraîne pas d'inquiétude dans la population ni de changements dans ses comportements. Cette situation est-elle à mettre sur le compte d'une transmission réellement faible de ce virus, sur une solide immunité croisée de la majorité ou sur un faible nombre de cas grave dans une population jeune peu utilisatrice de soins en absence d'atteinte importante?

## Liens d'intérêts

L'auteur déclare ne pas avoir de liens d'intérêts.
